# Structural Characterization of Lytic Transglycosylase SltB2 of *Pseudomonas aeruginosa*

**DOI:** 10.1021/acsomega.5c05747

**Published:** 2025-10-10

**Authors:** Vega Miguel-Ruano, María T. Batuecas, Elena Lastochkin, Teresa Domínguez-Gil, Rafael Molina, Shahriar Mobashery, Juan A. Hermoso

**Affiliations:** † Department of Crystallography and Structural Biology, Institute of Physical-Chemistry “Blas Cabrera”, 16379 Spanish National Research Council (CSIC), Serrano 119, Madrid 28006, Spain; ‡ Department of Chemistry and Biochemistry, 6111 University of Notre Dame, Nieuwland Science Hall, Notre Dame, Indiana 46556, United States

## Abstract

Lytic transglycosylases (LTs) belong to a family of enzymes that turnover the bacterial cell-wall peptidoglycan through a nonhydrolytic cleavage of the β(1–4) glycosidic bond, generating a hallmark 1,6-anhydromuramyl moiety in the reaction products. LTs are essential for numerous cellular processes, including cell-wall maturation, peptidoglycan recycling, cell division, and the assembly of multiprotein complexes. Their functional diversity underscores their biological significance. Family 3 LTs are distinguished by their EF-hand Ca^2+^-binding motif and are classified into two subfamilies. Subfamily 3B members, including *Pseudomonas aeruginosa* SltB2, possess a peptidoglycan-binding domain absent in subfamily 3A. In this study, we present the structural characterization of *P. aeruginosa* SltB2. The high-resolution crystal structure of SltB2 reveals a unique modular architecture shaped by the specific arrangement of its PG-binding domain and distinct differences in the organization of key residues surrounding the catalytic Glu residue compared to other family 3 members. A model of interaction between SltB2 and the peptidoglycan is proposed, which accounts for the enzyme’s tolerance to peptide stems and reveals particular features at site +2, due to the unique arrangement of the PG-binding domain, explaining its preferred exolytic activity. Comparative structural analyses of Family 3 LTs provide insights into substrate recognition and enzymatic function, advancing our understanding of bacterial cell-wall remodeling mechanisms.

## Graphical abstract

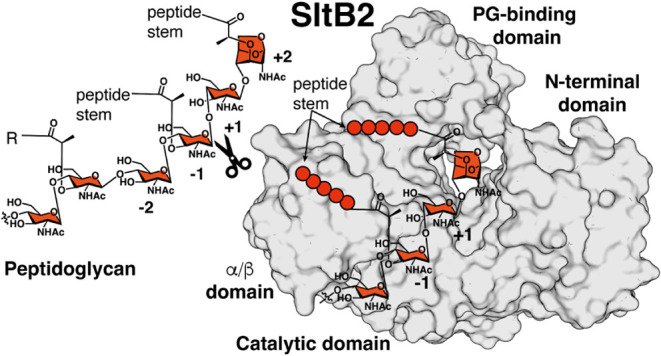

## 1 Introduction

The bacterial cell wall plays critical roles in defining the organism’s shape, ensuring survival in the face of turgor pressure, and facilitating cellular communication.^1,2^ This essential structure comprises a dynamic macromolecule called peptidoglycan (PG) as its major structural constituent. PG is a polymer consisting of repeating units of the disaccharide *N*-acetylglucosamine (NAG)-*N*-acetylmuramic acid (NAM), with a peptide stem attached to the NAM unit. The stem peptide is composed of l-Ala-d­(γ)-Glu-*m*DAP-d-Ala-d-Ala (*m*DAP for *meso*-diaminopimelic acid) in *Pseudomonas aeruginosa* and most Gram-negative bacteria. The neighboring peptide chains experience cross-linking, affording rigidity to the cell wall. PG synthesis involves numerous enzymes operating in both the cytoplasmic and periplasmic spaces. Among these enzymes, penicillin-binding proteins (PBPs) are prominent, playing a dual role in PG biosynthesis by mediating polymerization (in bifunctional class A PBPs) and/or cross-linking of the neighboring chains (in class A PBPs and monofunctional class B PBPs) through their transglycosylase and transpeptidase activities, respectively. In addition to PG synthesis, peptidoglycan hydrolases (also known as autolysins) are responsible for cell-wall remodeling. These proteins include endopeptidases and carboxypeptidases, as well as lytic transglycosylases (LTs).^1^ This last family represents a crucial class of autolysins with essential roles in diverse processes, including nascent PG sizing during cell-wall biosynthesis,^3^ PG recycling, and the release of signaling molecules associated with virulence^4^ and antibiotic resistance (recent reviews on this topic are available,^5–7^ as detected by phenotypic screens).^8^ Indeed, the composition of the muropeptide pool, generated during PG recycling, influences the expression of β-lactamases via the AmpR transcription factor.^9,10^ LTs are also important in cell division^11^ alone or through the incorporation of multiprotein structures into the cell wall,^12–14^ as well as in the sporulation and germination of Gram-positive spores.^6^ In contrast to glycoside hydrolases, LTs catalyze a nonhydrolytic cleavage at the β(1–4) glycosidic bond between NAM and NAG residues, producing the hallmark 1,6-anhydroNAM moiety in the product.^12^ The functional importance of LTs is underscored by their multiplicity in bacteria, with a myriad of reactions that they carry out on the cell wall, often with inherent redundancies, as observed in *E. coli* ^2^ or in *P. aeruginosa*.^15^ *P. aeruginosa* produces 11 LTs, with both membrane-bound (Mlt) and soluble (Slt) varieties. LTs are classified into six families according to their catalytic folds.^1,6,16^ Family 1 shares sequence homology with goose-type lysozymes and is subdivided into five subfamilies (1A–E) in *E. coli*: Family 2 includes MltA of *E. coli;* Family 3 contains consensus motifs similar to Family 1 but with insertions within the catalytic module;^6^ Family 4 comprises λ bacteriophage endolysins; Family 5 includes the PG chain-terminating enzyme MltG^3,17^ found in both Gram-negative and Gram-positive bacteria; and Family 6 includes the RlpA protein, which has a preference for the absence of the peptide stem.

Family 3 LTs are notable for their unique EF-hand motif, which binds a Ca^2+^ cation. This family is further divided into two subfamilies: subfamily 3A, which encompasses *E. coli* MltBs and *P. aeruginosa* SltB1, and subfamily 3B, which includes *P. aeruginosa* SltB2 and SltB3, as well as *Stenotrophomonas maltophilia* MltB2.^6^ Subfamily 3B of the LTs is distinguished by the presence of a putative peptidoglycan-binding domain (annotated as PG_binding_1, Pfam PF01471) at the C-terminal end, absent in subfamily 3A members like MltBs and SltB1 ^6^ (Figure 1). Recent structural studies have revealed the three-dimensional structures of *P. aeruginosa* SltB1 ^18,19^ and SltB3.^20^ Both proteins share a similar catalytic domain, along with an N-terminal and α/β domain.

**Figure 1:**
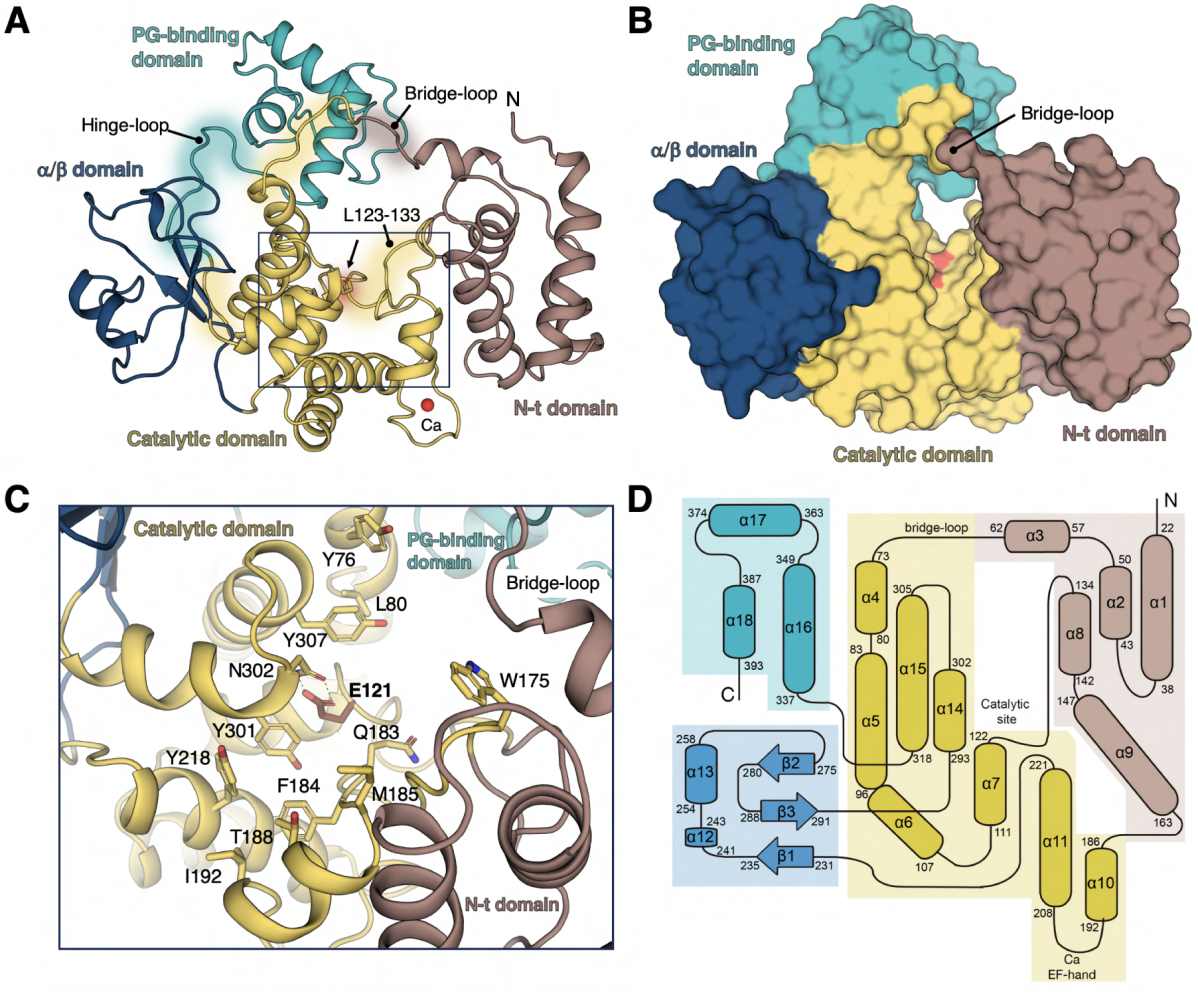
Crystal structure of *P. aeruginosa* SltB2. (A) Ribbon representation of the 3D structure of SltB2 with domains colored differently: N-terminal domain (brown), catalytic domain (yellow), α/β domain (dark blue), and peptidoglycan-binding domain (cyan). The catalytic Glu121 is shown as capped sticks and labeled. The Ca^2+^ ion in the EF-hand is represented as a red sphere. (B) The molecular surface of SltB2, color-coded as in panel A, with the catalytic site highlighted in red. (C) A detailed view of the SltB2 catalytic domain, with residues forming the catalytic groove shown as capped sticks. (D) Topographical diagram showing the secondary structure elements and domain organization of SltB2, with colors corresponding to those in the other panels.

We describe in the present report the structural characterization of SltB2 of *P. aeruginosa* for which the enzymatic reaction with the PG substrate was experimentally documented.^15^ SltB2 features the three modules observed in SltB3, but intriguingly, it exhibits a unique modular architecture not observed in other known structures of Family 3 LTs. Structural analysis and comparisons with related enzymes provide insights into substrate recognition and catalytic turnover by SltB2.

## 2 Methods

### 2.1 Production and Purification of SltB2

The cloning of the *sltB*2 gene (PA1171) and the purification of the corresponding gene product (SltB2) were reported previously.^15^

### 2.2 SltB2 Crystallization

SltB2 was concentrated to 6 mg/mL in 50 mM Tris, pH 7.4, 0.4 M NaCl, and 0.05% Brij-35. Crystallization was performed at 18 °C using the hanging-drop vapor-diffusion method by mixing 1 μL of protein with 2 μL of precipitant solution, which contained 20% (w/v) polyethylene glycol 4000, 0.1 M sodium acetate, and 8% (v/v) 2-propanol. Drops were equilibrated against 500 μL of the precipitant solution in the reservoir chamber. For cryoprotection, crystals were immersed in a solution containing 25% (w/v) polyethylene glycol 4000 without any additional components and then immediately flash-cooled in liquid nitrogen.

### 2.3 Data Collection, Phasing, and Model Refinement

The SltB2 data set was collected at 100 K with a PILATUS 6M detector on beamline XALOC of the ALBA Synchrotron (Barcelona, Spain). Crystals belong to the monoclinic P 2_1_ space group with one molecule in the AU (Table S1). Data processing and scaling were accomplished with XDS^21^ and AIMLESS.^22^ The SltB2 structure was solved by molecular replacement using the catalytic domain of the SltB3 structure (PDB code 5ANZ) as a partial model for search purposes. Searching using the complete SltB3 structure^19^ (94% sequence coverage with 50% identity) was unproductive, likely due to the dramatically different conformation found for the PG-binding domain. The rotational and translational searches were performed using PHASER^23^ and the primary obtained model was then subjected to iterative cycles of model building and refinement with COOT^24^ and PHENIX.^25^ Statistics for the crystallographic data and structure solutions are summarized in Table S1.

## 3 Results

### 3.1 Overall Structure of SltB2

The crystal structure of SltB2 from *P. aeruginosa* was determined at atomic resolution (1.70 Å) using the molecular replacement method, with the catalytic module of SltB3 (PDB code 5ANZ) serving as the initial model (see Methods and Table S1). A single chain was present in the asymmetric unit. The SltB2 structure exhibits a high-quality electron density map throughout the entire protein (residues 19–398) (Figure S1). The SltB2 structure adopts an annular arrangement of four different domains (Figure 1): (i) an N-terminal α-domain (residues 19–66, 129–173, Figure 1D) composed of five α-helices, (ii) a transglycosylase SLT domain (Pfam SLT_2 PF01464 ^26^ (residues 67–128, 174–226, and 292–323, Figure 1D) (iii) an α/β domain (residues 227–291), and (iv) a PG-binding domain (residues 324–398, Pfam annotated PF01471.^26^ The catalytic domain includes the catalytic Glu121 residue (Figure 1C) (as deduced by comparison with all the Family 3 LTs, Figure S2A) and the Ca^2+^ EF-hand motif, characteristic of Family 3 LTs (Figure 1A).

This domain organization resembles that of other Family 3 LTs from *P. aeruginosa* (SltB1 and SltB3)^18–20^ and *E. coli* (Slt35).^27^ Accordingly, Foldseek^28^ identifies the closest structural homologues of SltB2 as Family 3 LTs from *P. aeruginosa*, including SltB1 (PDB codes 4ANR and 5O8X, RMSD of 1.297 Å with 197 Cα atoms superimposed)^18,19^ and SltB3 (PDB code 5ANZ, RMSD of 1.155 Å with 282 Cα atoms superimposed),^20^ as well as *E. coli* Slt35 (PDB codes 1D0L and 1LTM, RMSD of 1.891 Å with 207 Cα atoms superimposed).^27,29^

The three pseudomonal enzymes of Family 3 LTs (Figure 2) share the catalytic, N-terminal α, and α/β domains; however, only SltB2 and SltB3 possess an additional PG-binding domain, a hallmark of the 3B subfamily of LTs. In line with this classification, SltB2 exhibits the highest sequence (50% identity, 67% identical or chemically similar) and structural homology with SltB3. However, the primary structural difference between SltB2 and SltB3 lies in the relative orientation of their PG-binding domains, which may reflect differences in substrate interaction or functional specialization. Comparison of the B-factor distribution in the three enzymes (Figure S2B–D) reveals that the highest B-factors in SltB2 are centered on two loops connected to the catalytic domain: the “bridge-loop” (residues 63–72), which links the first three helices to the catalytic domain, and a loop spanning residues 123–133. Additionally, to a lesser extent, high B-factors are observed in several loops within the α/β domain (residues 236–253, 264–275, 281–285), the PG-binding domain (residues 353–360 and 377–382), and the first two α-helices of the N-terminal domain (Figure 2B). In SltB1 and SltB3, the bridge-loop also contains high B-factors (Figure 2C,D). As detailed below, the mobility of the bridge-loop, positioned over the substrate-binding site, could affect its catalytic activity.

**Figure 2:**
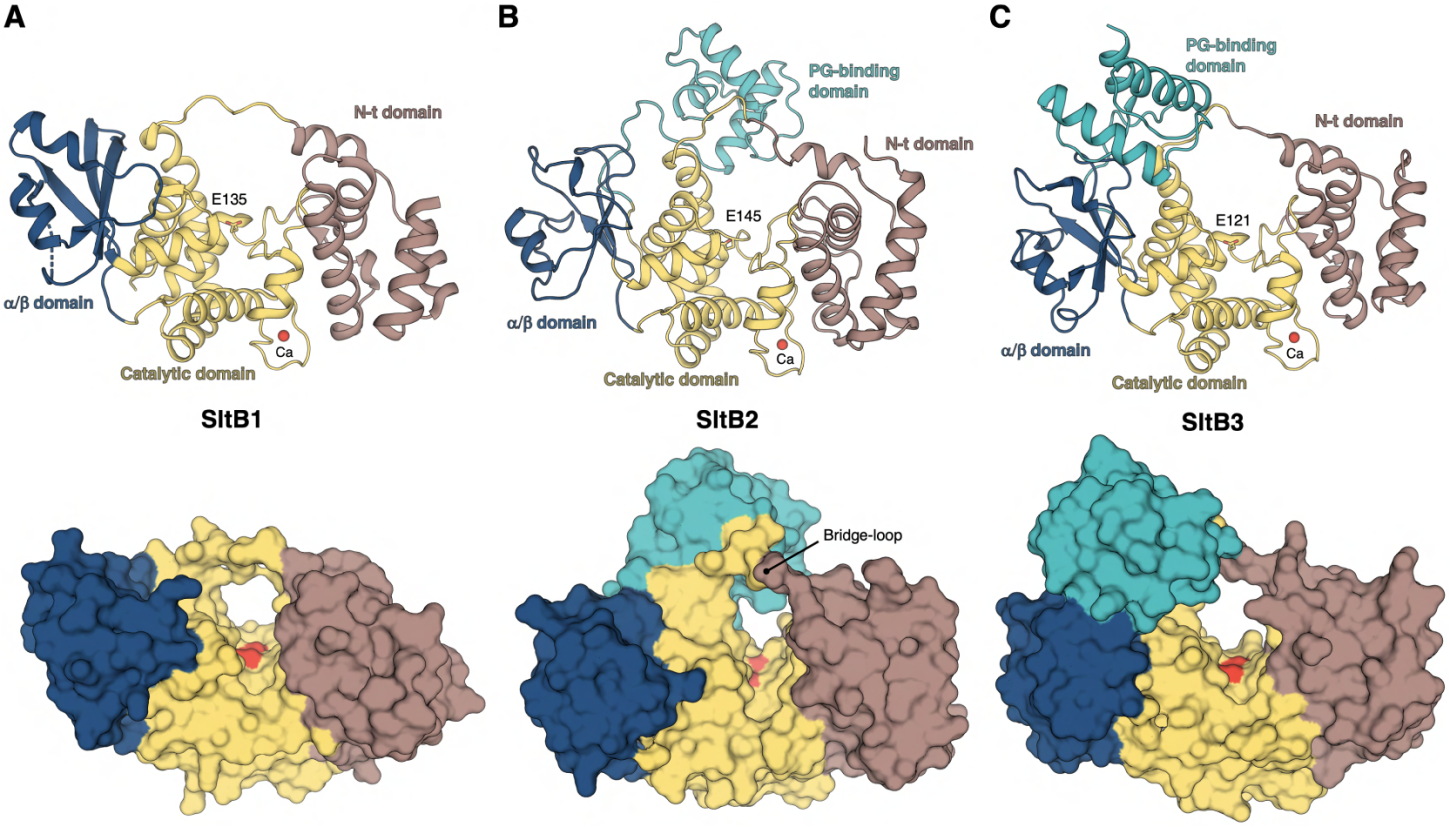
Modular arrangement in SltB1, SltB2, and SltB3 of *Pseudomonas aeruginosa*. Top: Ribbon representation of the three-dimensional structures with domains colored per the scheme of Figure 1. The catalytic glutamate is labeled and shown in capped sticks. The Ca^2+^ cation at the EF-hand is represented as a red sphere. Bottom: The solvent-accessible surface is depicted, with a red surface for the catalytic glutamate. (A) SltB1; (B) SltB2; and (C) SltB3. The three proteins are presented in the same perspective after superimposition of the catalytic domains.

### 3.2 The Catalytic Domain of SltB2

The catalytic domain of SltB2 is sandwiched between the N-terminal and α/β domains and contains the catalytic site and an EF-hand-like motif (Figure 1).^30^ The structure resembles that of SltB3 (RMSD of 0.54 Å for Cα atoms) and SltB1 (RMSD of 0.95 Å for Cα atoms) (Figure 3A). Sequence and structural alignment of SltB2 with these LTs (Figures 3A and S2A) reveals Glu121 as the catalytic residue involved in acid/base chemistry, positioned within a groove that serves as the substrate-binding site. As observed in other enzymes of this family, such as SltB1 and SltB3 of *P. aeruginosa* ^18–20^ or Slt35 of *E. coli*,^27^ this groove is primarily lined with aromatic and hydrophobic residues (Tyr76, Leu80, Trp175, Phe184, Met185, Thr188, Ile192, Tyr218, Tyr301, and Tyr307), which contribute to peptidoglycan recognition, along with the conserved Glu183 and Asn302 residues^20,27^ (Figures 1C and 3). Notably, SltB2 must retain peptide-binding regions accounting for the enzyme’s preference for substrates containing a peptide chain, as demonstrated in activity assays with peptidoglycan fragments.^15,27^ However, the most notable differences compared to other LTs in this family are observed at the +2 position (Figure 3B), where residue conservation is poorer, along with the presence of the PG-binding domain, tightly packed against the catalytic domain at this position. Besides, the completely visible bridge-loop in the electron density (Figure S1) partially covers the active site at this position (Figures 1B and 2).

**Figure 3:**
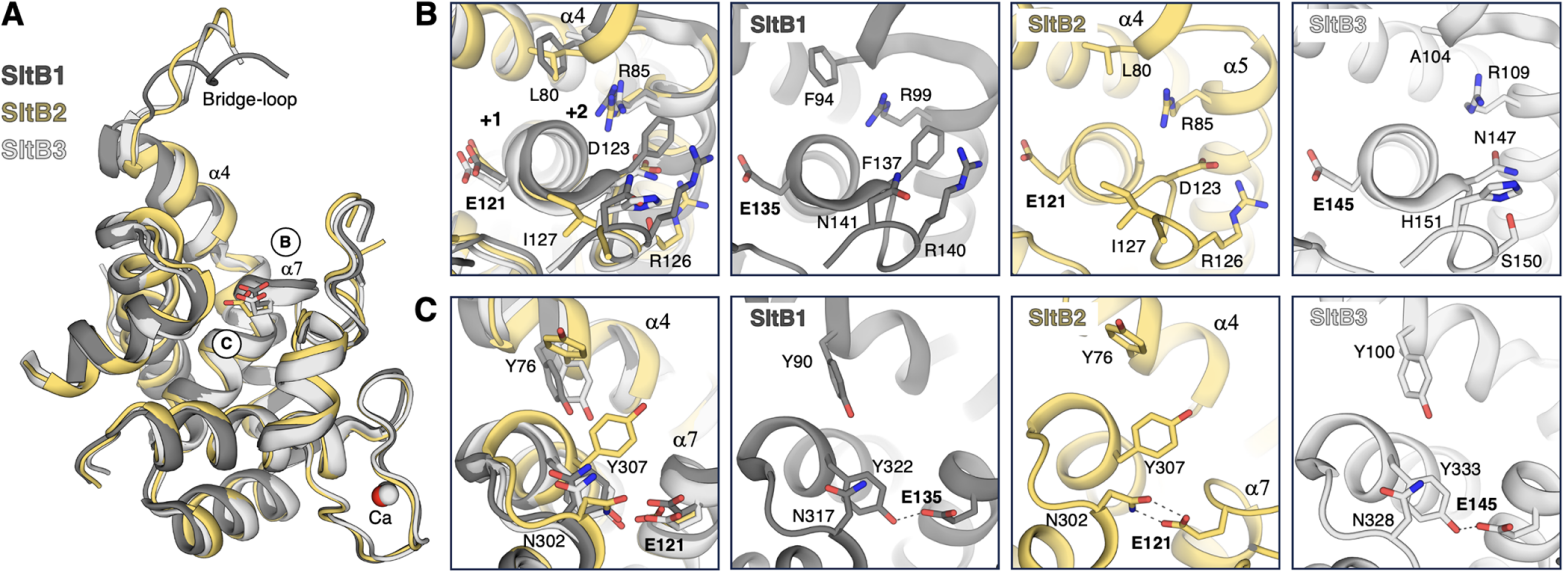
Catalytic-domain and active-site comparison among *P. aeruginosa* SltB1, SltB2, and SltB3. (A) Structural superimposition of the catalytic domains of SltB2 (yellow), SltB1 (dark gray), and SltB3 (white) is shown as ribbon representations. The catalytic residues—Glu121 (SltB2), Glu135 (SltB1), and Glu145 (SltB3)—are displayed as capped sticks, and Ca^2+^ ions are represented as spheres (5 o’clock). Two regions near the active sites are labeled **B** and **C** for expanded views in the corresponding **B** and **C** panels. (B and C) Detailed views of regions B (corresponding to substrate-binding subsites +1 and +2) and C, respectively. From left to right: structural superimposition of SltB1, SltB2, and SltB3, followed by the individual representations of SltB1, SltB2, and SltB3. Key residues are shown as capped sticks and labeled, with polar contacts depicted as dashed lines.

Interestingly, the crystallographic structure of SltB2 reveals distinct differences in the arrangement of key residues surrounding the catalytic Glu121 compared with SltB1 and SltB3 (Figure 3C). While the orientation of catalytic Glu121 in SltB2 aligns with that of SltB1 (Glu135) and SltB3 (Glu145) (Figure 3A), the side chains of residues around the catalytic one (Asn302, Tyr307, and Tyr76) in SltB2 adopt markedly different conformations compared to their counterparts in SltB1 and SltB3 (Figure 3C). In both SltB1 and SltB3, the catalytic glutamate forms a hydrogen bond with a tyrosine residue (Tyr322 in SltB1 and Tyr333 in SltB3) (Figure 3C), an interaction critical for catalysis as it maintains the proper positioning of the catalytic glutamate within the active site, as observed in *E. coli* MltE.^13^ In contrast, the catalytic Glu121 in SltB2 does not interact with its equivalent tyrosine (Tyr307), which presents a distinct conformer, positioning its hydroxyl group 7.5 Å away from it (Figure 3C). Furthermore, the side chain of Tyr307 in SltB2 occupies the position of Tyr100 in SltB3 (or Tyr90 in SltB1), which in turn forces a conformational shift in the corresponding tyrosine residue (Tyr76) in SltB2 (Figure 3C). Despite the absence of a hydrogen bond with Tyr307, Glu121 remains correctly positioned in SltB2 through a bifurcated hydrogen-bond interaction with Asn302 (Figure 3C), which adopts a conformation different from that in SltB1 or SltB3. It is worth noting that AlphaFold prediction for SltB2 shows an arrangement of the key residues similar to those observed in SltB1 and SltB3 (Figure S3), and the model exhibits a distinct conformation of the bridge loop, likely influenced by the conformational shift of Tyr76, resulting in a more closed catalytic groove.

As mentioned before, the catalytic domain of SltB2 contains an EF-hand-like motif positioned within an insertion between helices α10 and α11 (compared to the homologous catalytic domain of Family 1) (Figure 1). Like SltB1 and SltB3, the EF-hand in SltB2 deviates from the canonical EF-hand structure, containing 17 residues in the EF-hand loop instead of the typical 12. A Ca^2+^ cation is bound to this loop, coordinated by the side chains of four aspartate residues (Asp195, Asp197, Asp199, and Asp210), the main-chain oxygen atom of Arg201, and a water molecule that completes the pentagonal bipyramidal configuration (Figure S4). This coordination pattern and the amino acids involved are conserved in SltB3 and SltB1, except for the amino acid at position 201 (Arg in SltB2, His in SltB1, and Lys in SltB3), which contributes to the coordination via the main-chain carbonyl oxygen (Figure 4). The bidentate ligand normally found at position 12 in canonical EF-hands is located in SltB2 at position 16 (Asp225).^20^ Based on these characteristics, SltB2 can be classified as a class 4 EF-hand-like protein according to ref. 30.

**Figure 4:**
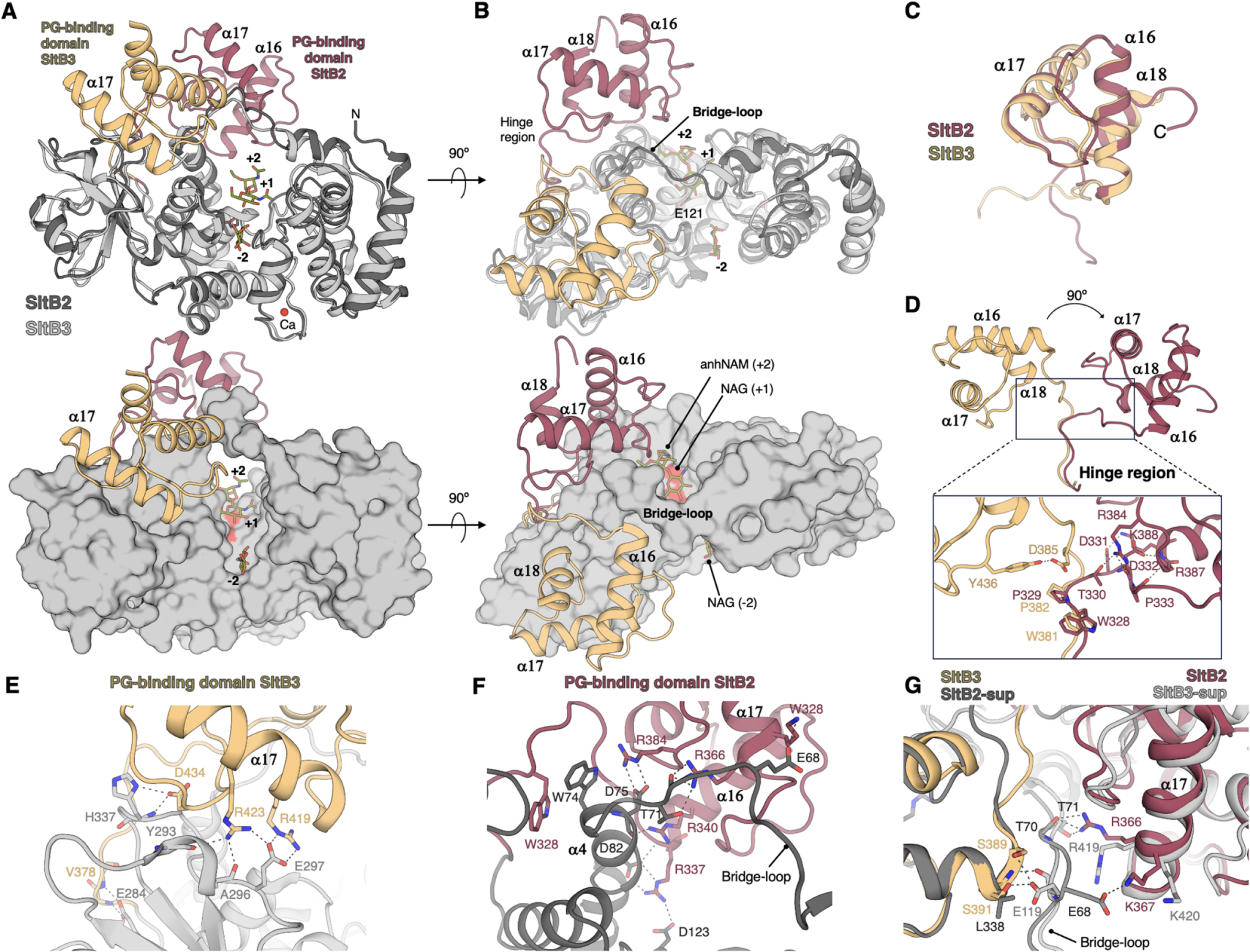
Structural comparison of the PG-binding domains in SltB2 and SltB3. The structure of SltB2 is shown in dark gray, and its PG-binding domain is highlighted in pink, while SltB3 is depicted in light gray, with its PG-binding domain in yellow. Key residues are represented as capped sticks. (A, B) Two perspectives (rotated 90°) of the structural superimposition of SltB2 and SltB3 (PDB code 5AO7 ^20^ crystallographic structures), shown in both cartoon and surface representations. The peptidoglycan fragment (NAG-anhydro-NAM) is represented as green sticks, and sugar subsites are labeled. (C) Superimposition of the PG-binding domains of SltB2 and SltB3. (D) Detailed view of the relative orientations of the PG-binding domains in SltB2 and SltB3, centered around the hinge region. (E, F) Stabilization of the different conformations found for PG-binding domains. (E) Interactions between the PG-binding domain and the α/β domain of SltB3. (F) Interactions between the PG-binding domain and the catalytic domain of SltB2. (G) Interactions between the PG-binding domains and the bridge-loop. The superposition of the PG-binding domain of SltB2 onto that of the SltB3 conformation (left) reveals the absence of a crucial serine (S391) in SltB2. Conversely, the superposition of the PG-binding domain of SltB3 onto the SltB2 conformation (right) highlights the absence of an important threonine (T71) in SltB3. In panels D-G, polar interactions are indicated by dashed lines.

### 3.3 Structural Comparison of the PG-Binding Domains in SltB2 and SltB3 and Functional Implications

SltB2, like its homologue SltB3 in the same subfamily, contains a type 1 peptidoglycan-binding domain (annotated as PF01471 ^26^ at its C-terminal end, which has been experimentally shown to bind peptidoglycan.^31^ The PG-binding domain of SltB2 adopts a characteristic three-α-helix fold spanning helices α16−α18 and four loops and contains two repeats of the conserved motif DGxxGxxTxxxPho (Pho for hydrophobic residues), important for recognizing a ligand of a repeating structure,^32^ spanning residues 358–369 and 382–393. However, the second repeat is degenerated due to the presence of an Ile instead of a Thr, which may impact ligand recognition. Structurally, the PG-binding domain of SltB2 is similar to those of ExeA and the Zn-dependent dd-peptidase (PDB: 1LBU, RMSD 0.769 for 29 Cα atoms). However, the composition of the critical residues is not conserved in SltB2, with modifications involving Glu372 (instead of Phe487 in ExeA) and Ile389 (instead of Thr503 in ExeA).

The PG-binding domain of SltB2 also resembles its counterpart in SltB3 (PDB: 5ANZ, RMSD 1.12 Å for 55 Cα atoms; Figure 4A–C), and the presence of this domain in these LTs causes structural changes in the bridge loop and the α/β domain (shortening the β-hairpin β2-β3) compared to SltB1 (Figure 2). Surprisingly, the PG-binding domain in SltB2 is rotated approximately 90° relative to its position in SltB3 (Figure 4D). This rotation is centered around the hinge loop connecting the catalytic and PG-binding domains, specifically within residues 328–330. Notably, Pro329, which is conserved in SltB3, appears to act as a hinge for this structural rearrangement (Figure 4D). Analysis of interactions with the PG-binding domain in the structures of SltB2 and SltB3 suggests that the displacement of this domain observed in SltB2 is driven by the formation of multiple new interactions. The PG-binding domain in SltB3 exhibits a single polar interaction with the hinge region (Figure 4D) (Asp385:Tyr436), whereas the domain in SltB2 forms multiple stabilizing contacts with residues in the hinge region (Figure 4D). Furthermore, the core of the PG-binding domain in SltB2 (including α16, α18, and their connecting loops) interacts with both the catalytic domain and the bridge loop (Figure 4F-G). Conversely, the PG-binding domain in SltB3 is positioned adjacent to the α/β domain, with numerous interactions among them (Figure 4E), while maintaining significant interactions with the bridge loop (Figure 4G). Also, strong interactions exist in SltB2 between the PG-binding domain and the catalytic domain, contributing to the stability of its rotated conformation. Specifically, three arginine residues within the PG-binding domain (Arg337, Arg340, and Arg384) form ion-pairing interactions with residues in the catalytic domain (Figure 4F). In contrast, the PG-binding domain forms strong interactions with the α/β domain (Figure 4E) in SltB3 through ionic interactions (Glu297:Arg419, Glu297:Arg423, and His337:Asp434) and hydrogen bonds (main chain of His337:Asp434, main chain of Tyr293:Arg423, main chain of Ala296:Arg423, and Glu284:main chains of Val378 and Val379). In conclusion, the different modular arrangements of the PG-binding domains in SltB2 and SltB3 are strongly stabilized through polar interactions with the other domains of the enzyme.

Given that the PG-binding domains of SltB2 and SltB3 exhibit a nearly identical fold (Figure 4C), one might speculate whether they could adopt the alternative modular arrangement observed in each LT. However, structural analysis suggests that such an exchange would be unfavorable. In the SltB2 modular conformation, the PG-binding domain of SltB3 would experience reduced stabilization due to the absence of Arg384 (Figure S5D), a key residue essential for maintaining the conformation observed in SltB2. Additionally, Asp75—responsible for a strong double ion-pairing interaction in SltB2—is not conserved in SltB3, where it is replaced by Lys. Similarly, if the PG-binding domain of SltB2 were placed in the SltB3 conformation, it would also lack key stabilizing interactions (Figure 5C). This is primarily due to the absence of Glu297 (replaced by Gly246 in SltB2), a critical residue in SltB3 that forms a strong ion-pairing interaction (Figure 5C). These observations strongly indicate that the distinct modular arrangements of the PG-binding domains in SltB2 and SltB3 are structurally constrained and not readily interchangeable.

**Figure 5:**
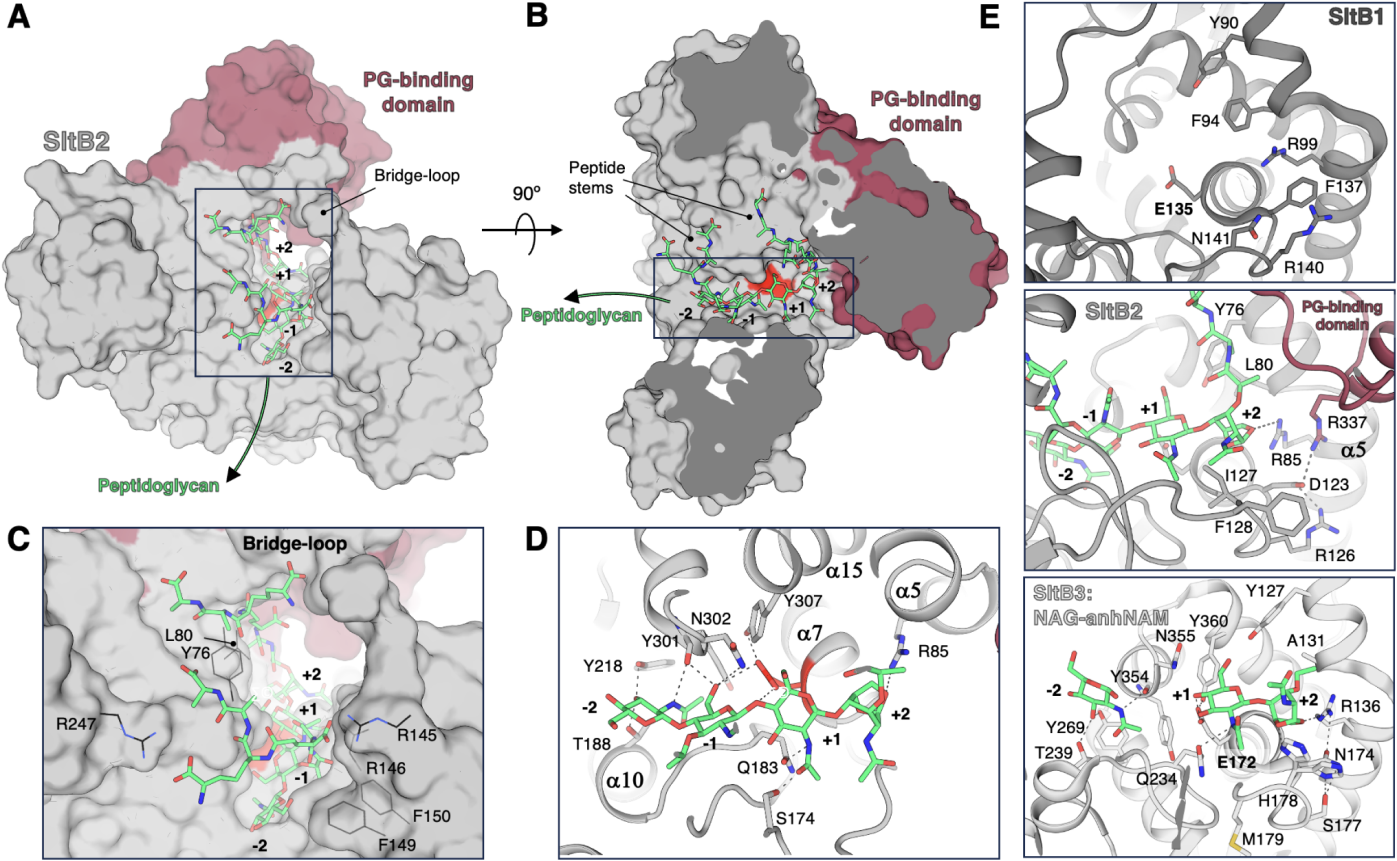
Computational model of the complex of PG with SltB2 for the exolytic reaction. (A, B) Rotated views (90°) of the computational model of SltB2 for the exolytic reaction with the substrate NAG-NAMpentapeptide-NAG-anhNAMpentapeptide. The SltB2 structure is shown as a gray surface, with its PG-binding domain highlighted in pink. The catalytic Glu121 is colored red, and the sugar subsites are indicated. The atoms of the peptidoglycan substrate are colored by atom type and shown as capped sticks (nitrogen in blue, oxygen in red, and carbon in green). Panels C and D provide detailed views of the boxed areas in A and B. (E) Zoomed-in views of the sugar subsite +2 in the structures of SltB1, SltB2, and SltB3. The SltB1 structure (PDB code 4ANR)^19^ is shown in dark gray, the SltB2 structure is represented in light gray, and the SltB3 structure in complex with NAG-anhNAMpentapeptide (PDB code 5AO7)^20^ is shown in white. Key residues are shown as capped sticks, and hydrogen-bond interactions are represented as dashed lines.

### 3.4 Modeling of Peptidoglycan Recognition by SltB2

The recognition of peptidoglycan in the SltB2 groove was modeled computationally (Figure 5), as all our trials to obtain crystal structures of the complexes of substrates and products failed. A computational fragment-based search using the FTMAP algorithm^33^ identified at least four sugar-binding subsites within the SltB2 catalytic groove, spanning positions −2 to +2 (with the site of reaction straddling subsites −1 and +1), similar to SltB3.^20^ Accordingly, a PG fragment was modeled incorporating four saccharide units and an anhydroNAM moiety at the terminal +2 position, consistent with its predominantly exolytic activity (>99%).^15^ The full-length pentapeptide was included in the model due to the enzyme’s preference for substrates with peptide stems^15^ and the conservation of peptide-recognition sites on the surface of the catalytic domain. Crystallographic complexes of SltB3 ^20^ and Slt35 ^27^ with PG analogs served as experimental references for this modeling.

The key residues that bind PG in SltB3 and Slt35 active sites are conserved in SltB2, as detailed below. The NAG moiety in the −2 subsite is predicted to be ensconced by the side chains of Tyr218 and Thr188, and the main chain of Tyr301 (Figure 5D). At the −1 position, NAM would interact with Asn302 and the catalytic Glu121, while the NAG at +1 engages with Gln183 and Glu121 (Figure 5D). The anhNAM moiety at +2 is nicely positioned in a hydrophobic pocket, similar to other LT complexes, with Ile127 and Phe128 from the 126–131 loop acting as platforms (Figure 5E). This region is largely devoid of interactions, with only Arg85 (Arg109 in SltB3) capable of forming a hydrogen bond. Our model also suggests specific recognition of stem peptides at positions −1 and +2 (Figure 5B). The carboxyl group of γ-d-Glu at the −1 NAM subsite is predicted to be stabilized by two conserved arginine residues (Arg145 and Arg146 in SltB2),^20,27^ while Arg247 from the α/β domain stabilizes the *m*-DAP of the peptide stem (Figure 5C). Similarly to the case of Slt35, SltB2 maintains a hydrophobic patch at the +2 position, which interacts with the aliphatic l-Ala-d-Glu chain through van der Waals interactions with the side chains of Tyr76 and Leu80 (Figure 5E).

The entry and binding of the peptidoglycan substrate, especially the peptide stems, into the SltB2 active site require some flexibility in the surrounding regions. Notably, the regions with the highest crystallographic B-factors in our structure—the bridge loop and the 126–131 region—frame the sugar backbone and the stem peptides in the substrate. Moreover, the binding of a cross-linked substrate appears highly restricted, which is consistent with the experimental outcome.^15^

## 4 Discussion

As stated earlier, *P. aeruginosa* has 11 LTs. Notwithstanding their overlapping activities,^15^ these genes have been conserved in the multitude of *P. aeruginosa* genomes that have been sequenced. It would appear that the gene products perform distinct functions, which serve as the selection pressure in the retention of the entire set of the 11 genes. The functions of these enzymes are not fully elucidated, but in some cases, they seem to serve as periplasmic hub proteins.^14,34,35^ A total of 71 periplasmic partner proteins to LTs have been identified, of which 24 are within the interactome of SltB2.^14^

We have undertaken to characterize all 11 LTs of *P. aeruginosa*. In this report, the crystallographic structure of SltB2 is described. The structure shares similarities with other Family 3 LTs (SltB1 and SltB3), including the presence of an N-terminal α-helical domain, an SLT domain with an EF-hand insertion coordinating a Ca^2+^ ion, and an α/β domain. The crystallographic structure of SltB2 reveals a general conservation of the residues that define the catalytic cavity but with differences in the composition of residues in the +2 subsite, as contrasted with the cases of SltB1 and SltB3. Interestingly, a rearrangement of key residues in the active site was observed in the structure. This reorganization involves a series of tyrosine residues that shift sequentially, ultimately altering the interaction of the key catalytic glutamate (Glu121) with Tyr307. This rearrangement also affects the conformation of the bridge loop. These structural differences, coupled with the observed B-factor distribution in the crystallographic structure (Figure S2), implicate the dynamic flexibility of the bridge loop in substrate recognition and catalysis.

Notably, SltB2 is closely similar to SltB3, a member of subfamily 3B, with both containing a peptidoglycan-binding domain. The PG-binding domain is widely distributed across both prokaryotic and eukaryotic proteins and is found in various bacterial enzymes, including LTs (e.g., SltB3 from *P. aeruginosa* (PDB: 5AO7), SleB from *Bacillus cereus* (PDB: 6CTI)^36^ and gp144 from the φ-KZ phage (PDB: 3BKV);^37^ amidases (e.g., AmpDh2 ^38^ and AmpDh3 ^39^); the ld-transpeptidase from *Stackebrandtia nassauensis* (PDB: 5BMQ); and other PG-processing proteins (e.g., CtpB protease from *Bacillus subtilis* (PDB: 4C2E)^40^ and EpsAB from *Vibrio vulnificus* (PDB: 4G54)^41^). A notable change in the orientation of this domain is observed between SltB2 and SltB3. In SltB3, the orientation of the PG-binding domain contributes to the formation of an open annular structure, approximately 18 × 24 Å in size, whereas in SltB2, the domain’s rotated conformation leads to a more restricted annulus, measuring 12 × 25 Å. This structural variation in the active-site cavity is clearly visible in the surface representations of the enzymes (Figure 2B,**C**). The tighter annulus in SltB2 likely impacts its enzymatic activity and substrate specificity. As highlighted by Lee et al., SltB1, SltB2, and SltB3 predominantly exhibit exolytic activity.^15^ The exolytic behavior of SltB2 is likely driven by the conformation of its PG-binding domain, which constrains the central opening of the enzyme. This limitation is likely at the root of the minuscule endolytic activity displayed by SltB2 (a mere 0.3% of the total activity with the sacculus as the substrate), as long PG strands are unable to pass through the narrowed annular space, restricting substrate access to the +2 position.

The absence of a PG-binding domain in SltB1 may reflect differences in cellular localization and secondary functions. Interestingly, SltB1 is known to interact with penicillin-binding protein PBP2 in a calcium-dependent manner.^19^ This interaction is consistent with the enzymatic activity described,^15^ where all three enzymes process tetrapeptide substrates (4p), supporting their potential role as PBP partners via the Ca^2+^ EF-hand. AlphaFold^42,43^ provides a structural model of the RodA:PBP2:SltB1 complex (Figures 6 and Figure S6) compatible with cell-wall synthesis and suggests that SltB1 interacts with PBP2 through its helical N-terminal domain and EF-hand loop. This model proposes a functional role for the calcium-binding EF-hand in mediating the interaction with PBP2, rather than serving a purely structural role as previously suggested in *E. coli*.^6^ EF-hands typically mediate calcium-dependent conformational changes that facilitate protein–protein interactions, enabling calcium signal-transmission pathways.^44^ The precise role of the EF-hand motif in these LTs remains an area of interest and warrants further investigation in the future.

**Figure 6:**
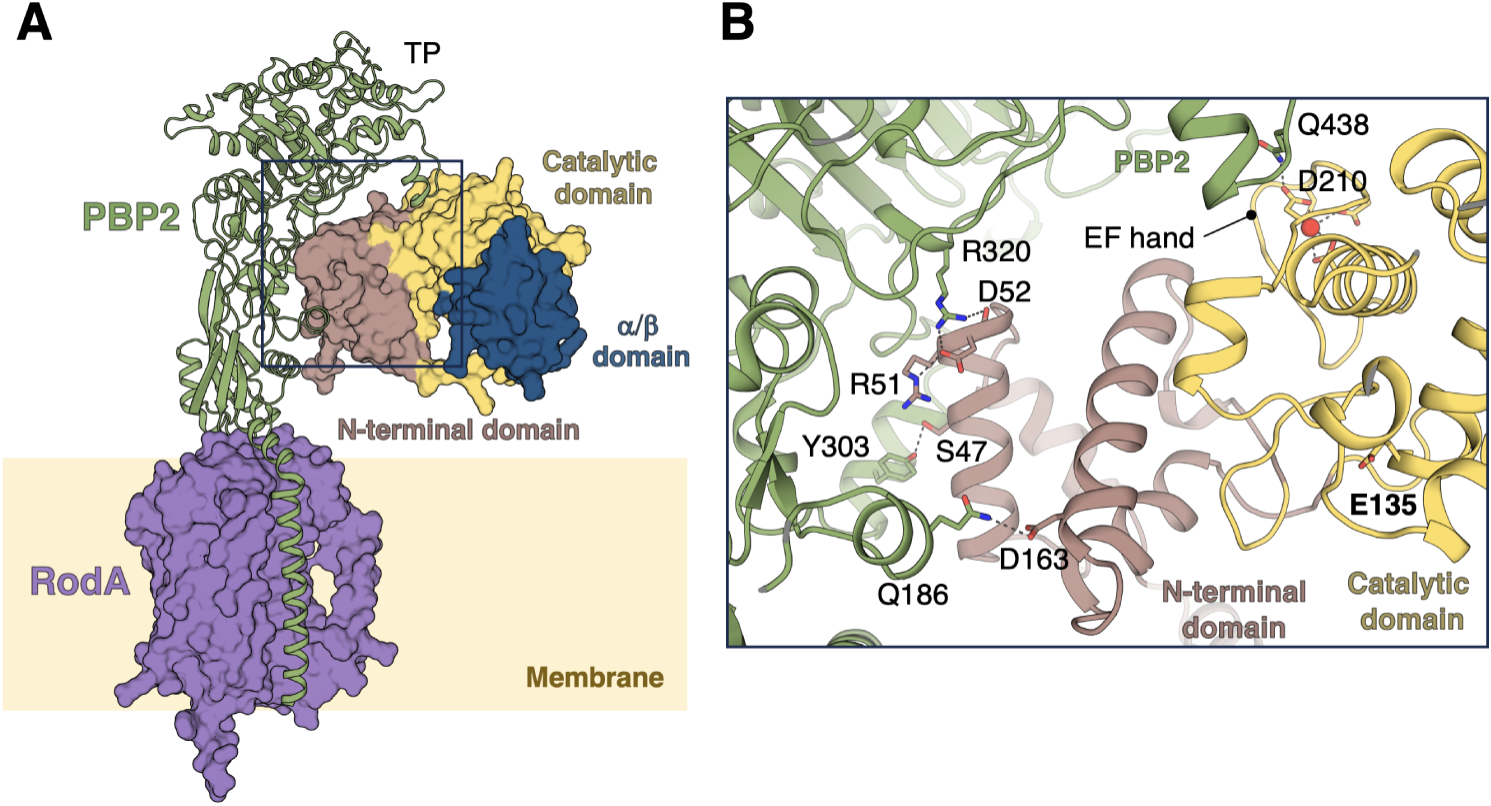
AlphaFold3 model of the *P. aeruginosa* RodA:PBP2:SltB1 ternary complex. (A) The predicted RodA (shown on the purple surface): PBP2 (in green ribbon): SltB1 (on the surface colored by domains) AlphaFold3 complex is depicted. The transpeptidase (TP) domain of PBP2 is labeled. (B) A detailed view of the PBP2:SltB1 interaction within the boxed area from panel A is shown. Key residues are depicted as capped sticks and predicted interactions are indicated with dashed lines. The Ca^2+^ ion is represented as a red sphere.

An intriguing three-dimensional structure of SltB1 by Domínguez-Gil et al.^18^ revealed a concatenated arrangement of SltB1, requiring the opening of the bridge loop that connects the catalytic domain to the N-terminal domain. The possibility of an open-close conformational transition of the catalytic site, mediated by this bridge, has also been reported for other Family 3 LTs.^45^ For such a structural arrangement to occur, the three α-helices of the N-terminal domain (α1–3) must reposition, which generally requires weak interactions between these helices and the rest of the domain. While hydrophobic interactions predominantly stabilize these helices in SltB1 and SltB3, the scenario is markedly different in SltB2, where polar interactions play a role (Figure 2B). These include ion pairing between Arg145 and Asp64, as well as Arg131 and Asp56. Additional hydrogen bonds link α1 and α2 to α9, and a disulfide bridge anchoring helices α1 and α8 seems to be the key point restricting conformational mobility. The lower B-factors observed in this region for SltB2, compared to SltB1 and SltB3 (Figure S2), support the notion of a more rigid N-terminal domain of SltB2. The concatenated conformation of SltB1 has been proposed to serve a regulatory function, as this inactive structural state disrupts its catalytic activity. Such a conformation appears less likely for SltB2 due to its enhanced structural rigidity. This raises an interesting question: could SltB2 employ an alternative regulatory mechanism through the distinct conformational dynamics of its PG-binding domain? The structural divergence observed between SltB2 and its homologues suggests that regulatory control in these enzymes may not be solely dependent on N-terminal domain flexibility but could also involve modulation via domain structural arrangements or transcription regulation. This hypothesis opens new avenues for understanding how structural plasticity contributes to the regulation of LT activity among different family members.

## Supplementary Material



## Notes

Figures of the electron-density map of SltB2; comparison of active sites and B-factors among SltB1, SltB2, and SltB3; AlphaFold-predicted model of SltB2; structural analysis of EF-hand domains in SltB1, SltB2, and SltB3; analysis of the stabilization of peptidoglycan-binding domain conformations in SltB2 and SltB3; AlphaFold model of the RodA:PBP2:SltB1 complex; and the Crystallographic Data Table (PDF)
