# Supplementary material for: Structural Characterization of Lytic Transglycosylase SltB2 of Pseudomonas aeruginosa
Source: ACS Omega. 2025 Oct 10;10(41):48385–94. doi: 10.1021/acsomega.5c05747 (PMC12547810; doi:10.1021/acsomega.5c05747)
Supplement: Supplementary file 1 [file ao5c05747_si_001.pdf]

Supplementary data

Structural Characterization of Lytic Transglycosylase SltB2 of *Pseudomonas aeruginosa*

Vega Miguel-Ruano[1,#], María T. Batuecas[1,#], Elena Lastochkin[2], Teresa Domínguez-Gil[1], Rafael Molina[1], Shahriar Mobashery[2,*] and Juan A. Hermoso[1,*]

[1]Department of Crystallography and Structural Biology, Institute of Physical-Chemistry "Blas Cabrera", Spanish National Research Council (CSIC), Madrid (Spain).

[2]Department of Chemistry and Biochemistry, University of Notre Dame, Notre Dame, Indiana 46556, United States.

[#] Both authors equally contributed to this work.

*To whom correspondence should be addressed. E-mail: mobashery@nd.edu and xjuan@iqf.csic.es

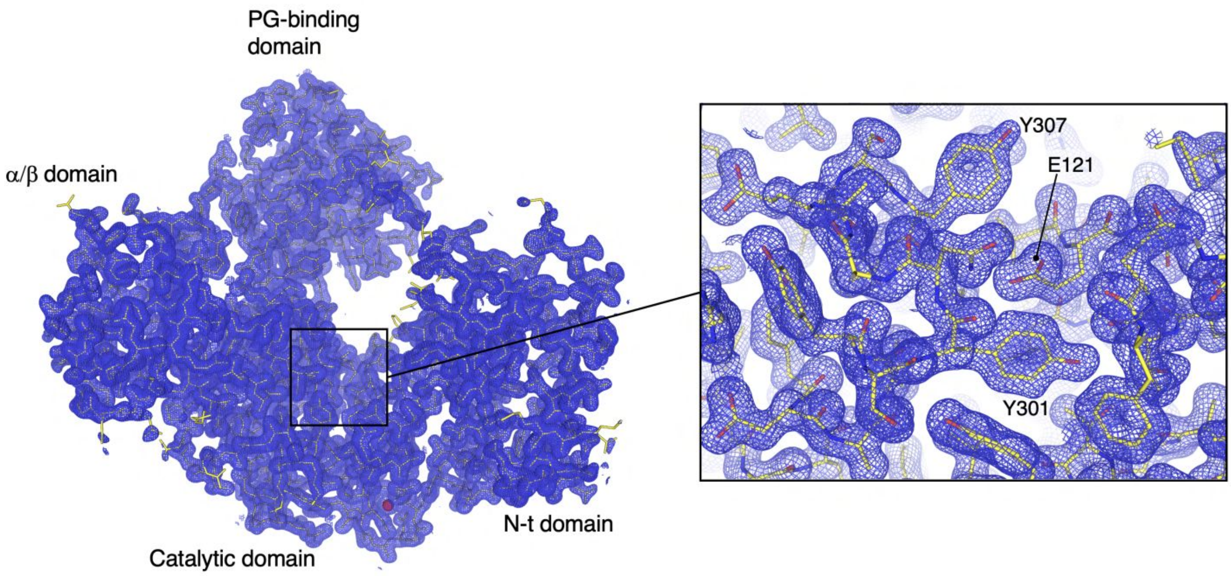

Figure S1. **Electron-density map of *P. aeruginosa* SltB2.** The $2F_o$-F_c map is contoured at 1 σ and is represented as a blue mesh. The SltB2 protein is shown as yellow-capped sticks, with the Ca^{+2} ion is represented by red sphere. The different SltB2 domains are labeled. The right panel offers a detailed view of the boxed active-site region.

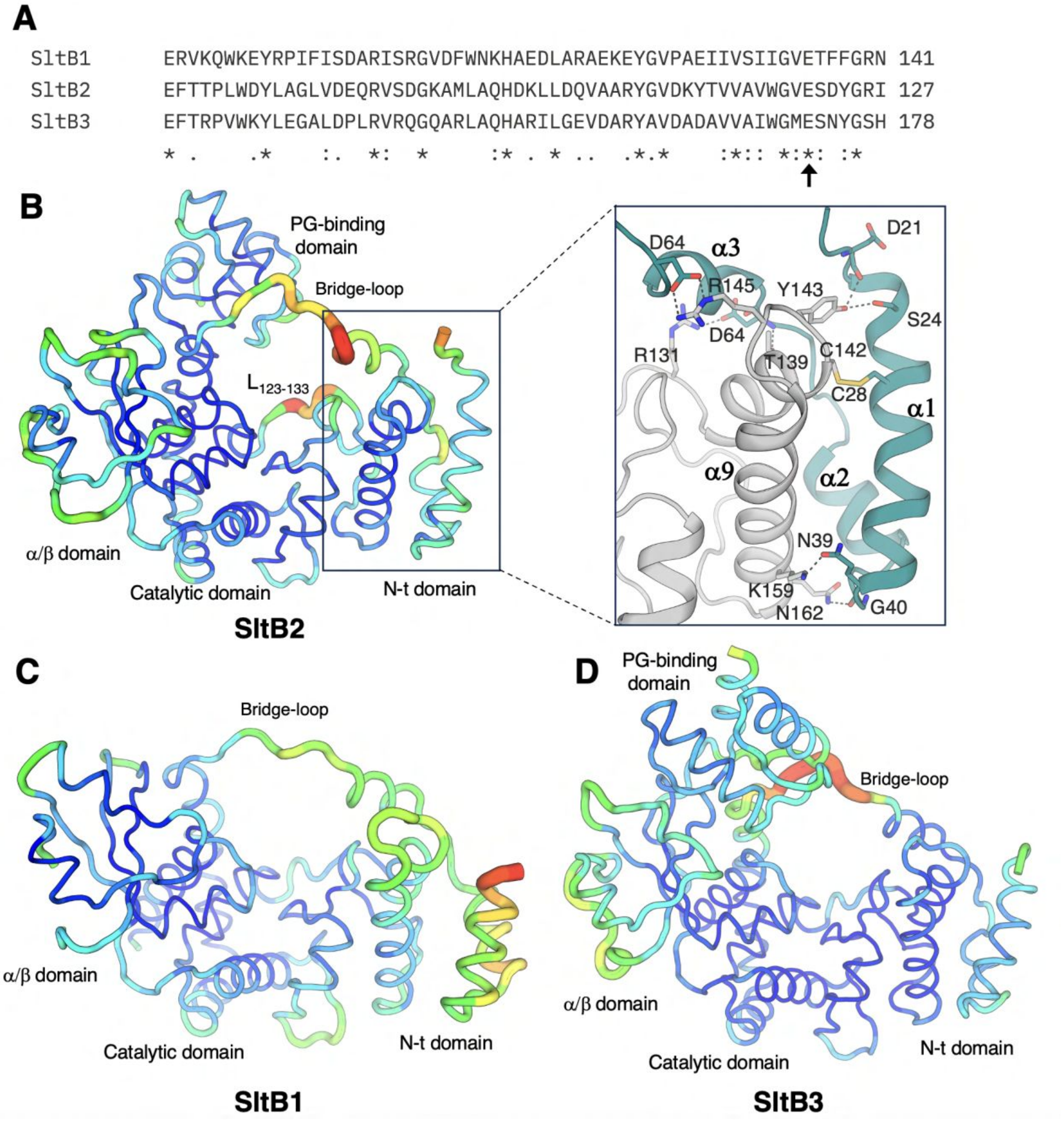

Figure S2. **Active site and B-factor comparison among SltB1, SltB2 and SltB3. (A)** Clustal Omega [1]sequence alignment of the active sites of SltB1, SltB2, and SltB3. The conserved catalytic glutamic acid (Glu121 in SltB2) is marked with an arrow. **(B-D)** B-factor putty representations of the crystallographic structures of SltB2 **(B)**, SltB1 (PDB code 4ANR [2]) **(C)**, and SltB3 (PDB code 5AO7 [3]) **(D)**. The structures are displayed as ribbon models, color-coded according to calculated B-factors. Regions with higher B-factors are shown in orange to red with a wider tube, while regions with lower B-factors appear in shades of blue and a narrowed tube. **(B)** B-factor representation of the SltB2 structure is given. The right panel provides a zoomed-in view of the stabilization of α1-3 helices (in blue) through interactions with the rest of the N-terminal domain (in gray). Interacting residues are shown as sticks and labeled.

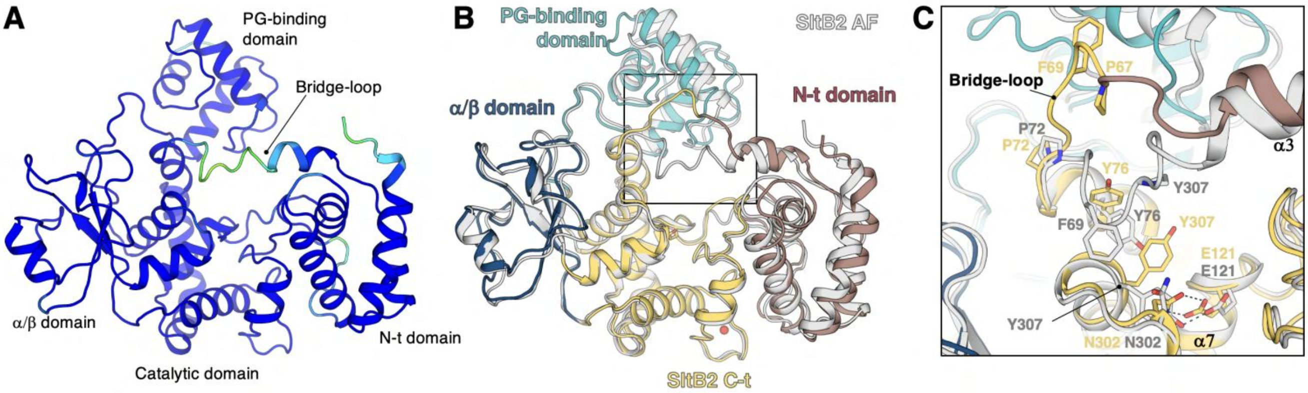

Figure S3. **AlphaFold3-predicted model of *P. aeruginosa* SltB2. (A)** AF3 [4]model of SltB2, color-coded by pLDDT **(**predicted local distance difference test) score. **(B)** Structural comparison between the crystallographic structure of SltB2 (colored by domains as in Figure 1) and the AF3 model (gray). A boxed region highlights differences in the bridge-loop. **(C)** Close-up view of the structural variations in the active site and bridge-loop between the crystallographic structure and the predicted model. Polar contacts are indicated in dashed lines.

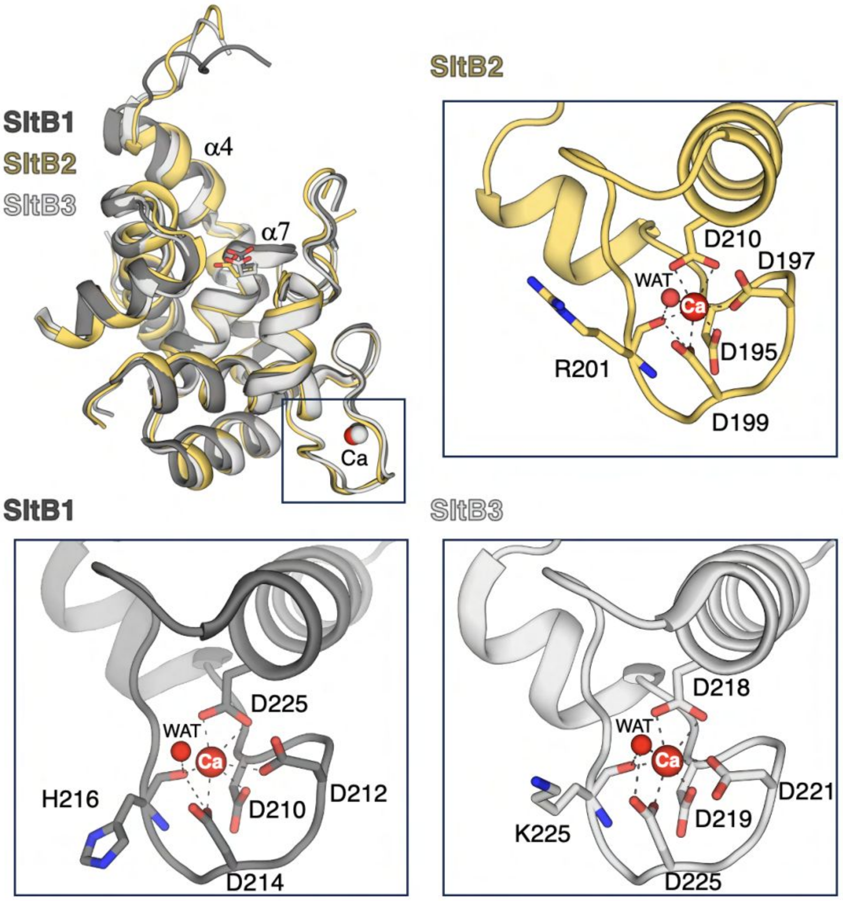

***Figure S4*: Structural analysis of the EF-hand in SltB1, SltB2 and SltB3.** Superimposition of the catalytic domains and the EF-hands: SltB1 in dark gray, SltB2 in yellow, and SltB3 EF-hand in white. Calcium ions are shown as red spheres for SltB2 and white spheres for SltB1 and SltB3. Water molecules are represented as red spheres. Boxed regions provide detailed views of calcium-ion coordination in the EF hand. **Top-right:** Calcium-ion coordination in SltB2 involves seven interactions with four aspartic acids, one water molecule, and a carbonyl group from the protein backbone. **Bottom**: calcium-ion coordination in SltB1 and SltB3.

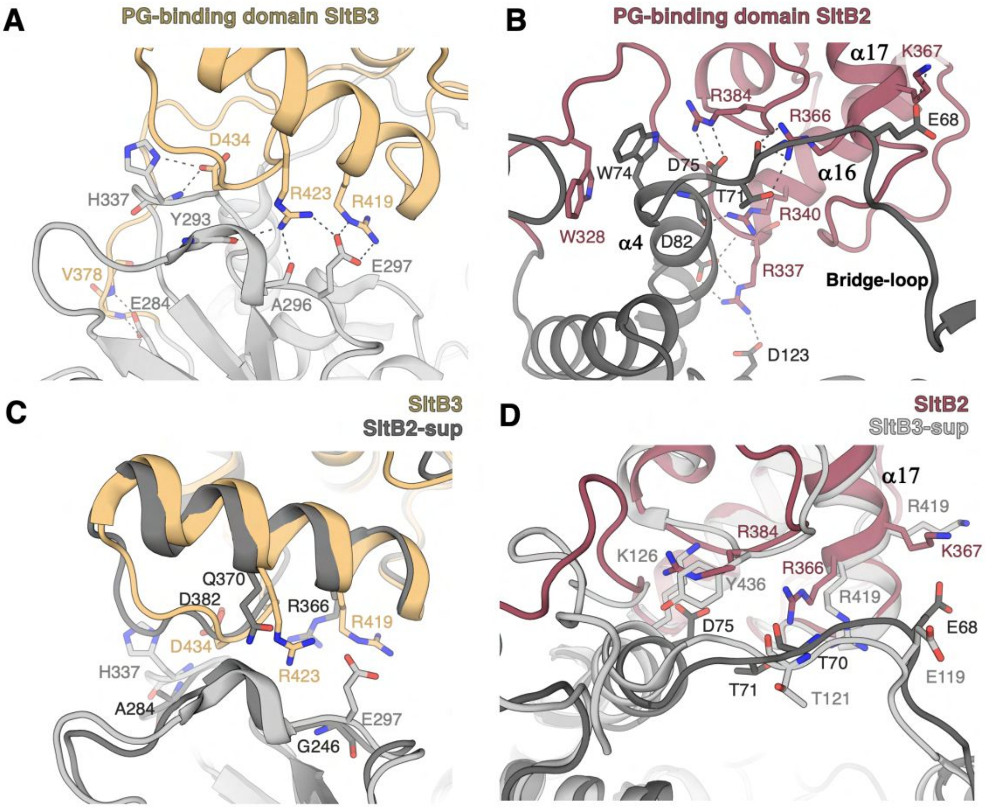

Figure S5. **Stabilization of the different conformations found for PG-binding domains in *P. aerugin*osa SltB2 and SltB3.** The structure of SltB2 is shown in dark gray, and its PG-binding domain is highlighted in pink, while SltB3 is depicted in light gray with its PG-binding domain in yellow. Key residues are represented as capped sticks, and polar interactions are indicated by dashed lines. **(A)** Interactions between the PG-binding domain and the α/β domain of SltB3, and **(B)** those between the PG-binding domain and the catalytic domain of SltB2. Are shown. **(C)** Superimposition of the PG-binding domain of SltB2 onto the SltB3 conformation and **(D)** that of the PG-binding domain of SltB3 onto the SltB2 conformation are depicted.

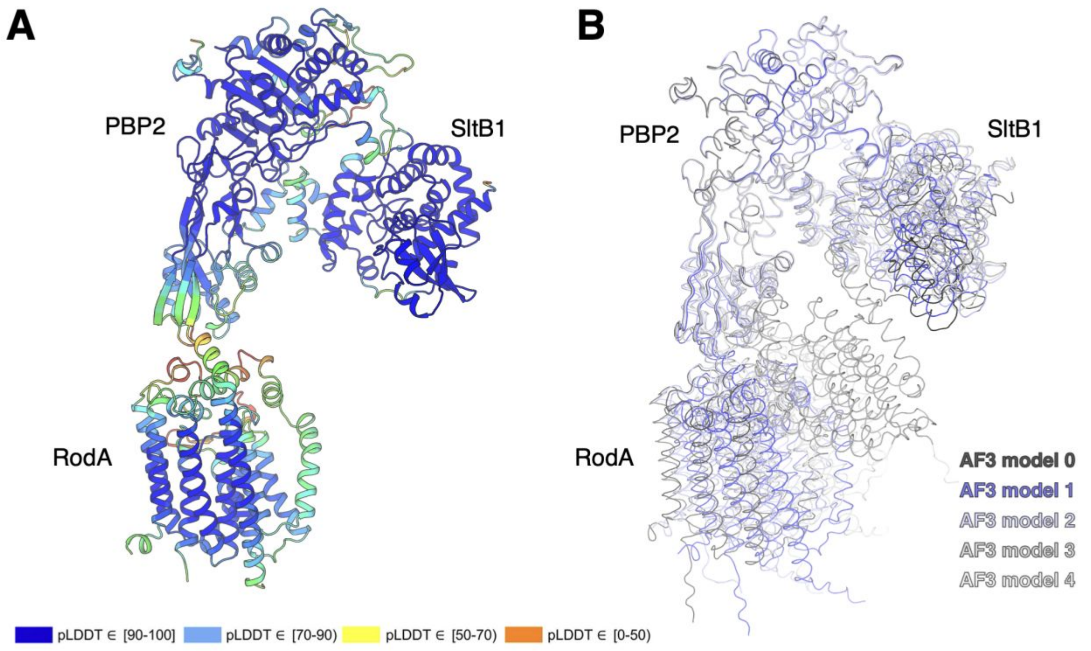

Figure S6. **AlphaFold3 model of the *P. aeruginosa* RodA:PBP2:SltB1 complex. (A)** Cartoon representation of the RodA:PBP2:SltB1 complex, with confidence levels indicated by a color gradient of pLDDT values. **(B)** Structural superimposition of five AF3 models, shown in varying shades of blue and gray.

Table S1.* Crystallographic data collection and refinement statistics

**Data Collection**[a]	**SltB2**
Wavelength (Å)	0.979
Space group	P 2_1
Cell dimensions	
a, b, c (Å)	46.97, 42.40, 93.58
β (°)	95.65
Resolution range (Å)	46.56-1.70 (1.73-1.70)
Unique reflections	36814 (1926)
Completeness (%)	99.0 (99.3)
Multiplicity	6.6 (6.9)
$CC_{1/2}$	0.99 (0.70)
R_{pim}	0.04 (0.44)
Avg. I/σ (I)	13.0 (2.3)
Refinement statistics	
Resolution range (Å)	46.56-1.70
Rwork/Rfree	0.16/0.19
No. atoms	
Protein	2913
Water	404
Others	1
Root-Mean-Square Deviations	
Bond length (Å)	0.011
Bond angles (deg)	1.63
Ramachandran favored/outliers (%)	97.1/0.3
Residues in the AU	379
PDB code	7QVD

[a]Values in parenthesis are referred to the highest-resolution shell indicated.

References

[1] Sievers F, Wilm A, Dineen D, Gibson TJ, Karplus K, Li W, et al. Fast, scalable generation of high-quality protein multiple sequence alignments using Clustal Omega. Mol Syst Biol 2011;7:539. https://doi.org/10.1038/msb.2011.75.

[2] Nikolaidis I, Izoré T, Job V, Thielens N, Breukink E, Dessen A. Calcium-dependent complex formation between PBP2 and lytic transglycosylase SltB1 of Pseudomonas aeruginosa. Microbial Drug Resistance 2012;18:298–305. https://doi.org/10.1089/mdr.2012.0006.

[3] Lee M, Domínguez-Gil T, Hesek D, Mahasenan K V., Lastochkin E, Hermoso JA, et al. Turnover of bacterial cell wall by SltB3, a multidomain lytic transglycosylase of Pseudomonas aeruginosa. ACS Chem Biol 2016;11:1525–31. https://doi.org/10.1021/acschembio.6b00194.

[4] Jumper J, Evans R, Pritzel A, Green T, Figurnov M, Ronneberger O, et al. Highly accurate protein structure prediction with AlphaFold. Nature 2021;596:583–9. https://doi.org/10.1038/s41586-021-03819-2.
